# Hidden labor, monitoring, and exit burdens after hospital operational AI deployment: implications for resource-constrained Chinese hospitals

**DOI:** 10.3389/fmed.2026.1985884

**Published:** 2026-10-09

**Authors:** Xieyi Wang, Hezhuo Wang

**Affiliations:** 1 Ningbo No. 6 Hospital, Ningbo, China; 2 The Affiliated People’s Hospital of Ningbo University, Ningbo, China

**Keywords:** cost incidence, digital health, health services research, model governance, organizational workload, technology implementation

## Abstract

Hospital operational artificial intelligence (AI) can reduce visible task time, but postdeployment affordability also depends on work transferred to staff, technical teams, budgets, and vendors. This focused structured narrative Mini Review examined hidden labor, monitoring and maintenance, and exit or replacement burdens after real hospital AI deployment, with implications for resource-constrained Chinese hospitals. Reconciliation of retained candidates produced a 23-record analytic inventory, including 15 reports grouped into 12 primary deployment clusters; other records supplied adjacent, contextual or governance evidence. Exhaustive coverage is not claimed. Evidence was classified by burden domain, bearer, and three outcome levels: observed work, labor value or opportunity cost, and budget or cash expenditure. Human verification, correction, adaptation, data upkeep, and coordination persisted in several deployments, although one controlled study found no significant increase in review burden. Monitoring required continuing ownership and remediation capacity. Direct operational-AI permanent-exit evidence remained limited to one hospital lifecycle case. Chinese reports identify workflow responsibilities but do not establish AI lifecycle affordability in resource-constrained hospitals. The proposed Pilot, Scale, Modify, Share, and Stop framework comprises locally testable management propositions, not validated thresholds or Chinese cost estimates. Prospective studies should measure burdens across clinical, administrative, technical, financial, vendor, and regional actors.

## 1 Introduction

Hospital operational artificial intelligence (AI) is promoted to reduce documentation time and support scheduling, coordination and patient flow. A large language model used for nursing handover across three Taiwanese hospitals shortened documentation time, while a UW Health pragmatic trial found benefits from ambient documentation support (1, 2). Yet a rapid review found only six eligible real-world ambient-scribe studies; productivity, cost, quality and safety evidence remained sparse (3). Visible gains matter where staff time is scarce, but focal-task evaluation may miss verification, correction, training, coordination, hosting, support and governance elsewhere.

This review uses cost incidence descriptively to ask which actor carries a resource requirement, not as a formal economic incidence analysis. A 2026 review found that only 28% of 117 AI economic evaluations reported implementation costs and 57% reported operating costs; an adjacent hospital decision-support review identified labor as the largest implementation-cost category (4, 5). The relevant bearers include clinical and administrative staff, information technology (IT) and data teams, management and finance, vendors, and regional or regulatory organizations. Postdeployment studies show that work can move across these groups (6–8). Observed work must also be separated from labor value and booked cash expenditure. A saved minute is not a budget saving unless it changes overtime, staffing, throughput or another resource that the hospital can release or redeploy.

The review asks: How should evidence on the distribution of hidden labor, monitoring, and exit-related burdens after hospital operational AI deployment inform decisions in resource-constrained Chinese hospitals? Operational AI is defined here as AI embedded in hospital documentation, scheduling, coordination, information, patient-flow or related administrative workflows. An LMIC scoping review found only 10 real-world studies and limited cost-effectiveness evidence, underscoring the risk of importing implementation claims without local measurement (9). China is the intended decision context. Retained Chinese reports identify operational review and institutional reporting responsibilities. The remaining gap concerns transferable lifecycle resource and cost measurement in resource-constrained hospitals, rather than an absence of Chinese deployments.

## 2 Review approach and analytic frame

This focused structured narrative review used AI-assisted web searches, including site-restricted searches for PubMed/PMC and publisher pages, Chinese-language discovery and reference following. Searches were conducted on 28 August 2026 and updated on 1 September. English/Chinese publications were eligible, without a lower publication-date limit. Previously identified records were also considered; X10 was drawn from an existing literature library and documented in the original 28 August candidate ledger. During retained-candidate reassessment, XW and HW independently screened records; XW extracted information and HW checked it. Uncertain classifications or disagreements were referred to a third expert for independent judgment.

During revision, retained candidates were reassessed using the review's L1 definition of observed work or workflow responsibility. Core eligibility required real hospital/health-system operational use, a reported postdeployment activity or responsibility, and an identifiable bearer. Activities included human review, integration, upkeep, oversight or transition; deployment, task routing or efficiency gains alone were insufficient. Quantified costs, large samples and full-text access were not prerequisites; vendor ties were caveats. This reassessment corrected inconsistent earlier selection decisions; the criteria were not preregistered. Supplementary Material 1 documents the methods; Supplementary Tables S2, S3 provide record-level decisions and available search records. The retained search records document the searches performed but do not provide a complete list of retrieved records or the original screening total. Exhaustive coverage is not claimed.

The original 18 records comprised nine core/companion reports representing seven deployment clusters, one secondary-core report, one supplementary implementation report, three adjacent, three contextual and one governance record. The submitted matrix omitted the supplementary report already cited in the manuscript. Revised selection yields 23 records: 15 primary/companion reports in 12 clusters, three adjacent, four contextual and one governance record. Sections S7 and S15 of Supplementary Material 1 separate original accounting from evidence-based selection changes. X08 remains contextual because its accessible abstract establishes task routing but not a specific burden-domain activity. UW Health, UCI and Shanghai each contribute a paired report family. Table 1 maps C1–C12. “Strict core” denotes a burden-focused analytic subset, not a quality grade. Background references are separate; no pooled estimate is presented.

**Table 1:** Burden and bearer map of 12 primary deployment clusters selected for detailed synthesis.

Setting and operational task	Design	Hidden labor	Monitoring/maintenance	Exit/replacement	Main bearers	Outcome level	Direction and principal limitation
C1. Taiwan, three hospitals; nursing handover (1)	Multisite implementation	Review, correction and confirmation	Not directly measured	Not reported	Nurses; hospital finance	L1, L2, limited L3	Time saving and positive estimated labor value; no demonstrated cash release
C2. UK NHS; digital command centre (12)	Mixed-method evaluation	Adaptation and data-quality work	Continuing data and organizational work	Not reported	Staff; IT/data; management	L1	Quantitative benefit uncertain; AI-specific attribution incomplete
C3. USA, UW Health; ambient documentation (2, 6)	Pragmatic trial plus implementation monitoring	Onboarding, review and remediation	Dashboards, weekly review, template redesign, training, license tracking	Not reported	Clinicians; IT/data; management; vendor	L1	Task and well-being benefits with recurring monitoring; one vendor-linked deployment
C4. USA, UCI Health; ambient documentation (10, 11)	Content analysis plus interviews	1,804 edits and contextual correction	Not measured	Not reported	Clinicians	L1	Correction content observed; time and net burden not measured
C5. Germany; EHR documentation copilot (18)	Non-randomized controlled pre-post study	Senior review and revisions	Not measured	Not reported	Clinicians	L1	Efficiency improved; no significant between-group increase in revision trajectory
C6. England; integrated and standalone ambient scribes (7)	Longitudinal qualitative evaluation	Templates, validation, copying and speech adaptation	Integration and vendor-interface work	Lock-in and continuity concerns	Staff; IT/data; management; vendor	L1	Architecture-dependent burden; primary and secondary care mixed
C7. Southeast Asian government hospital; information chatbot (8)	Longitudinal qualitative lifecycle case	Data upkeep, approvals, training and coordination	Licenses, secure hosting, support and reallocation	Cost-benefit review, sunset and transition	Staff; IT/data; management; vendor	L1, limited L3	Direct exit evidence; single anonymous tertiary-hospital case
C8. Pediatric scheduling, Israel (19)	Retrospective pre/post	Chief-resident review and adjustment	Not separately costed	Not reported	Chief residents; scheduling team	L1	Equity and earlier publication are distinct from roster-preparation effort; edit logs unavailable; vendor ties
C9. Ambient documentation, New York (20)	Six-month implementation evaluation	Training and integration	Ongoing technical support	Not reported in abstract	Multidisciplinary team; clinicians	L1	Official abstract only; efficiency benefits do not quantify support costs
C10. Japan; discharge summaries (13)	Small implementation study	Therapist verification and correction	Not separately costed	Not reported	Therapists	L1	Exploratory; retained review is not proof of net burden increase
C11. China; equipment priorities (17)	Campus comparison	Engineer adoption recording and decisions	AI-support costs not quantified	Not reported	Clinical engineers	L1	Device maintenance costs are not AI maintenance costs; tertiary setting
C12. China; Shanghai outpatient ordering (15, 16)	Cohort and subsequent trial	Physician checking/approval of orders	Not separately costed	Not reported	Physicians	L1	Same institutional program, different periods; patient fees are not AI ownership cost

C1–C12 correspond to Supplementary Tables S1, S2. “Strict core” denotes this purposively selected analytic subset, not a quality grade or exhaustive count. Fifteen reports form 12 clusters: UW Health, UCI and Shanghai each have two linked reports. The Shanghai reports evaluate successive periods of the same institutional program, not identical samples. L1, observed work or workflow responsibility; L2, labor value or opportunity cost; L3, budget or cash expenditure. Limited L3 denotes expenditure categories or partial operating costs, not a complete budget-impact evaluation. Equipment-maintenance or patient-care spending is not classified as AI-support expenditure. EHR, electronic health record; IT, information technology.

Evidence was classified along three dimensions. Hidden labor includes verification, correction, training, coordination, data upkeep and workflow adaptation. Monitoring and maintenance include dashboards, data-quality checks, revalidation, remediation, license oversight, hosting and vendor interfaces. Exit and replacement include reassessment, termination, migration and fallback work. Five bearer groups were mapped across three outcome levels: L1 observed work or workflow responsibility, L2 labor value or opportunity cost, and L3 budget or cash expenditure. Qualitative and implementation studies can identify mechanisms and responsibility at L1, but they do not by themselves establish L2 or L3 magnitude. Recent reports and large tertiary or academic systems predominate, limiting stability and numeric transfer.

## 3 Hidden labor after deployment

Human review and correction remained part of routine use in several deployments. Nurses using the Taiwanese handover system had to inspect, correct and confirm generated content before submission (1). At UCI Health, analysis of 200 real encounters involving 73 clinicians and 33 specialties identified 1,804 word-level edits to ambient-AI drafts. The edits involved examinations and orders, symptoms, medications and diagnoses (10). Companion interviews attributed modifications to transcription errors, speaker attribution, omissions, excessive certainty, clinical context, and billing or legal requirements (11). These studies establish the existence and content of correction work, but not its duration or net workload. Edit counts should not be converted into labor hours without time measurement.

System architecture influenced where hidden labor appeared. In the English regional ambient-scribe evaluation, integration with the electronic health record required standardization, template development and validation. Standalone use was easier to start but transferred information through manual copying, while clinicians also adjusted how they spoke so that the system captured their reasoning (7). The Bradford command-centre evaluation reported staff adaptation and data-quality work within a broader digital program, although the AI component could not always be separated from the platform as a whole (12). In the Southeast Asian chatbot case, department representatives uploaded and updated content in addition to their existing work, while implementation teams handled approvals, training, communication and license reallocation (8). Automation thus redistributed tasks among frontline staff, technical teams and managers rather than establishing that work had disappeared.

Retained reports describe therapist verification and correction in Japan (13) and physician approval of AI-generated pre-examination orders in Shanghai (15, 16). Chinese engineers recorded model-recommendation adoption and maintenance actions (17). These establish activities and bearers, not incremental workload or AI cash costs; research-period recording does not establish a permanent AI-support task. The Brazilian abstract describes request routing across staff groups (14), but does not establish a specific verification, upkeep or oversight activity. It is contextual, not a core burden deployment. Its small sample and vendor affiliations limit interpretation. Routine professional work cannot all be attributed to AI.

The direction of net burden was not uniform. Task-level gains in Taiwan and UW Health counter a purely burden-centered interpretation (1, 2). A 12-month controlled pre-post study in German ophthalmology retained senior-physician review but found no significant between-group difference in revision trajectories, while documentation efficiency improved among AI users (18). A pediatric scheduling comparison retained human review and reported improved equity and earlier roster publication. Mean on-call shifts per physician did not decline; this measures allocated clinical work, not time spent constructing or checking rosters (19). Human-edit logs were unavailable. Its vendor relationships warrant caution, not exclusion solely on that basis. AI may therefore reduce a task while leaving review requirements or other workload in place.

Evidence becomes thinner when hidden work is translated into economic outcomes. The Taiwanese study estimated 474 to 981 h saved per month across the three hospitals, calculated a labor value for that time, and reported monthly generative-AI operating costs of US$1,871 to US$2,464 (1). The authors did not treat estimated labor value as direct budget savings. That distinction is decisive for hospital management. Capacity becomes financially releasable only if time gains change paid overtime, staffing requirements, service throughput or the allocation of constrained personnel. Across the core studies, L1 evidence on tasks and bearers was much stronger than L2 valuation, while L3 evidence on cash consequences for clinical and administrative hidden labor was largely absent.

## 4 Monitoring and maintenance as recurring capacity

Production monitoring functioned as an organizational service rather than a one-time check. UW Health used dashboards, multidisciplinary governance, weekly review and license-utilization tracking (6). Bradford and the hospital chatbot case required attention to data quality, use, support and resource allocation (8, 12). An abstract-accessible six-month New York health-system report identified integration, training, interoperability, analytics and technical-support needs (20). For this revision, only its official abstract and bibliographic record were accessible; they did not provide quantified support costs, and no full-text cost-extraction claim is made. These activities suggest a need for operational ownership. Treating monitoring as generic IT overhead can obscure whether existing staff can absorb marginal work without displacing services. A Hebei mixed-platform report describes routine institutional summaries (21); it is retained as context because separable AI-specific postdeployment work is unclear.

Synthesis-level evidence supports this lifecycle view. A systematic review of 25 clinical-AI frameworks found that planning was covered more often than later action, with little LMIC representation (22). A healthcare MLOps review identified monitoring, retraining, infrastructure, human resources and finance as linked operating domains (23). Interviews with 21 experts further described a “responsibility vacuum” in which monitoring and maintenance were fragmented, inconsistently performed and undervalued (24). These sources explain why ownership and capacity require explicit design, but none supplies a transferable cost estimate.

Monitoring plans should specify indicators, review frequency, responsible staff and escalation authority. Technical availability alone does not establish useful uptake or workflow fit. A dashboard can flag a problem but cannot supply the staff time, vendor cooperation or authority needed to resolve it. This is an implementation inference, not a measured cost estimate.

Monitoring also produced protective value. At UW Health, a template change was associated with a fall in ICD-10 documentation accuracy from 79% to 35%. The monitoring process identified the decline and prompted template redesign, targeted training and changes to onboarding (6). The event should not be generalized as an expected effect size for other tools. It demonstrates a management mechanism: monitoring consumes resources because detection must be followed by investigation and remediation, but the relevant counterfactual includes silent degradation. Evidence from four deployed predictive models and 17 internally developed radiology algorithms provides adjacent support for paired technical-operational ownership, scheduled monitoring, dashboards and escalation processes (25, 26). These adjacent sources clarify organizational practice but do not increase the count of operational-AI core deployments.

Recurring dependencies extend beyond performance metrics. The English ambient-scribe study described trade-offs between integrated and standalone products and an immature, volatile vendor market (7). UW Health purchased its system as software as a service, while vendor employees participated as authors in the monitoring report; the study reported no vendor funding (6). The hospital chatbot required licenses, secure cloud hosting and support manpower, and its team reallocated unused licenses as use patterns changed (8). These findings support scrutiny of contract renewal, data access, interoperability and vendor response, but none provides a complete total-cost estimate.

Four questions follow before scale-up. Who monitors which clinical, operational and utilization indicators? Who has protected time and authority to respond? Which budget pays for hosting, revalidation, retraining and remediation? Which thresholds trigger modification or a stop review? Existing studies answer parts of the first question and identify several bearers. They rarely quantify opportunity cost or separate AI-specific expenditure from shared infrastructure. Monitoring capacity should therefore be recorded locally at L1, valued at L2 where feasible, and linked to actual L3 payments rather than represented by a single unqualified cost figure.

## 5 Exit and replacement burdens

Exit capacity is part of affordability because a successful pilot does not guarantee that the system remains useful, supportable or distinct from later alternatives. The NASSS framework links sustainability and abandonment to the technology, value proposition, organization, wider system, and adaptation over time (27). This non-AI-specific foundation supports treating exit as a management outcome, while the English ambient-scribe evaluation identified vendor-market volatility and potential lock-in (7). These concerns were not monetized, but they show why reversibility should be examined before procurement and scale-up.

Exit need not indicate technical failure: funding, vendor support, alternative functions or workflows may change. A proposed planning checklist should cover interfaces and data, fallback and retraining, contractual obligations, staff communication and safety responsibilities. These categories organize local preparation; the present evidence does not quantify their combined burden or establish that each occurs in every withdrawal.

The strongest direct lifecycle evidence came from the Southeast Asian government-hospital chatbot. The system launched hospital-wide in December 2023 and was discontinued in February 2025. During operation, departments maintained content, the implementation team completed regulatory and organizational approvals, and the hospital carried license, secure-hosting and support requirements. Low use and overlap with other systems prompted a cost-benefit review. The innovation team recommended sunset and transition to existing systems, and management accepted the recommendation (8). This sequence links recurring burden to an actual stop decision. It does not quantify exit cost or establish a threshold that other hospitals should copy.

Adjacent evidence confirms that withdrawal itself creates work. A multisite NHS survey after removal of a chest-radiograph AI tool documented logistical burden, although its response rate was 21.4% and the task lies outside the strict operational scope (28). A large health-system governance case reported 10 deployments and two retirements but did not quantify the resources used (29). Contracts and pilot plans should therefore assign responsibility for data export, decommissioning, retention, vendor cooperation, workflow continuity and a funded transition period. No included study validates universal exit criteria, and regional-platform responsibilities remain a major evidence gap.

## 6 Implications for resource-constrained Chinese hospitals

Reassessment of mainland-China reports identified engineers’ maintenance-priority workflow and Shanghai's physician-reviewed outpatient ordering program (15–17). These reports document postdeployment responsibilities but do not establish an increase in net workload. The equipment study's maintenance-cost ratios concern medical devices, not the cost of maintaining AI. Patient expenditure and waiting time likewise do not measure hospital AI ownership cost. These tertiary settings provide insufficient evidence to assess lifecycle affordability in resource-constrained hospitals. Hebei's institutional reporting account remains contextual evidence (21). Diagnostic-imaging implementation interviews provide additional context (30), as does administrators’ individual LLM use rather than formal organizational deployment (31). Wages, staffing, infrastructure, procurement and vendor support may change burden magnitude. The following decision gates are proposals for local testing, not validated rules.

A **Pilot** gate should establish a local denominator. Hospitals can record baseline task time, required review and correction, training and coordination, IT support, license and hosting payments, and the person accountable for each item. Logs should preserve L1 activity without assuming that saved minutes have a monetary value. L2 valuation can then distinguish protected staff time from work absorbed on top of existing duties. L3 reporting should record marginal payments and any realized change in overtime or staffing. The Taiwanese evidence is useful here because it separates time, estimated labor value and operating cost rather than collapsing them (1).

A **Scale** decision should require evidence that visible gains relieve a binding operational constraint and that monitoring capacity will grow with use. License utilization, error and correction patterns, technical incidents, workflow transfer, and staff groups carrying uncompensated work should be reviewed together. A **Modify** decision is appropriate when monitoring identifies a remediable template, workflow or training problem and the hospital can staff the response, as illustrated by the UW Health monitoring case (6). A **Share** option may place hosting, monitoring or specialist support in a regional platform, but only if financing, data governance, escalation and accountability are explicit. Shared infrastructure can reduce duplication while also transferring work to an actor whose capacity is rarely measured in the present evidence.

A Stop review should be activated by locally declared combinations of persistent low use, functional redundancy, unresolved risk, vendor discontinuity or recurring burden that exceeds the value of continued use. The chatbot case shows how low use and redundancy can enter a cost-benefit reassessment, while governance and offboarding studies identify transition tasks that must be allocated (8, 28, 29). These are candidate criteria, not validated cut-offs. Predefined export formats, contract termination rights, record-retention rules, workflow fallback and a transition budget make the stop option credible.

Research in China should prospectively observe these decisions in county, municipal and resource-constrained tertiary hospitals. Priority designs include time-driven activity measurement of review and coordination, multicentre comparison of vendor and hosting arrangements, budget-impact studies that separate wage valuation from marginal cash, and lifecycle studies that document modification or exit. Samples should include nurses, administrators, IT and data personnel, finance staff, procurement teams and regional platform managers. Without these perspectives, studies may report tool performance while leaving the distribution of organizational work unobserved.

## 7 Discussion and conclusion

The reviewed evidence supports the existence and distribution of postdeployment work more strongly than its monetary magnitude. Correction and adaptation persisted in several cases, yet the German controlled study found no significant increase in review burden. Monitoring required resources while detecting correctable degradation. Direct operational-AI permanent-exit evidence came from one case. Limitations include purposive burden-focused selection, incomplete retrieval and selection documentation, heterogeneous designs, concentration in large tertiary systems and recent publications, incomplete AI-specific attribution, and sparse L2/L3 measures. Chinese tertiary evidence is not a resource-constrained cost estimate. The 12 clusters are not representative, and their frequencies cannot estimate prevalence. Abstract-level evidence supports only activities explicitly reported there.

These limitations preclude pooled cost estimates and strong cross-setting prediction. They permit a provisional account of who performs continuing work and identify variables for prospective local measurement; they do not establish net savings or cost-effectiveness.

Hospital operational AI should be assessed beyond task-time gains alone. Resource-constrained hospitals can use the proposed framework to identify continuing work, assign monitoring responsibility and consider modification or exit before scaling. Figure 1 connects task benefits, redistributed burdens and outcome levels to locally testable decisions. Its value is to structure measurement and deliberation, not to prescribe universal thresholds or predict financial returns.

**Figure 1:**
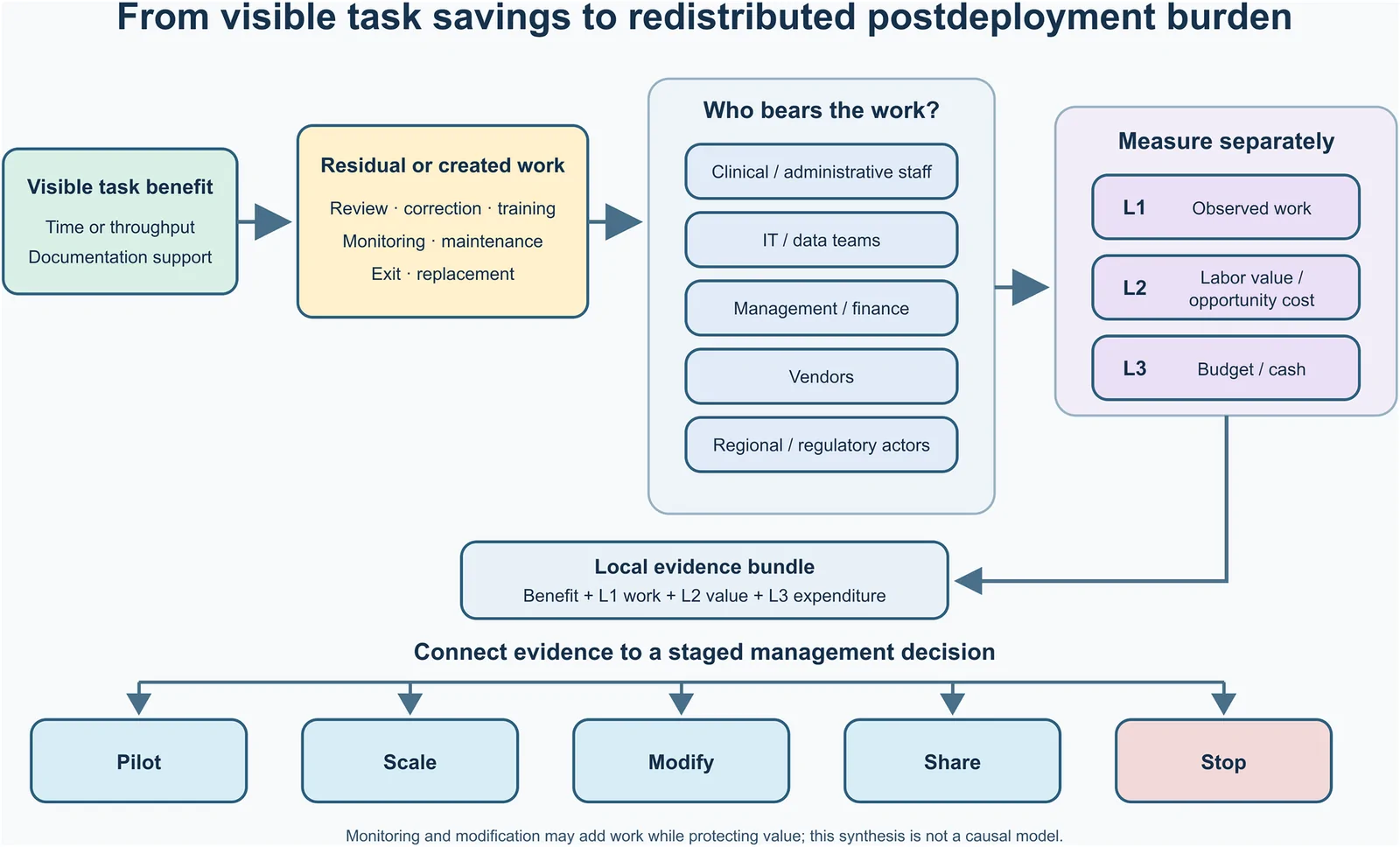
From visible task savings to redistributed postdeployment burden. Visible task benefits may coexist with residual or newly created work. Hospitals should allocate that work across clinical and administrative staff, IT/data teams, management and finance, vendors, and regional actors; measure it separately at L1 workflow, L2 labor-value and L3 budget levels; and connect the evidence to Pilot, Scale, Modify, Share or Stop decisions. Monitoring and modification can add work while protecting value. The figure is a synthesis proposed by this review, not a validated causal model.
