# Supplementary material for: Hidden labor, monitoring, and exit burdens after hospital operational AI deployment: implications for resource-constrained Chinese hospitals
Source: Front Med (Lausanne). 2026 Oct 9;13:1985884. doi: 10.3389/fmed.2026.1985884 (PMC13691341; doi:10.3389/fmed.2026.1985884)
Supplement: Supplementary file 1 [file Table1.docx]

Supplementary Material 1

Search, screening, source verification and evidence classification

# S1. Review question and design

How should evidence on the distribution of hidden labor, monitoring, and exit-related burdens after hospital operational AI deployment inform decisions in resource-constrained Chinese hospitals? Operational AI was limited to AI embedded in hospital documentation, scheduling, coordination, information, patient-flow, or related administrative workflows. Pre-procurement assessment, algorithm-development cost, laboratory-only performance studies, patient out-of-pocket cost, and purely clinical AI without separable operational consequences were outside the strict core.

This is a focused structured narrative review, not a systematic review. “Strict core” denotes the 12 primary deployment clusters used to examine burden-bearing mechanisms, not a quality tier or an exhaustive denominator. No meta-analysis, formal certainty grading or validated risk-of-bias score is claimed.

# S2. Sources, dates and restrictions

| **Item** | **Documented approach** |
| --- | --- |
| Initial search | 28 August 2026; actual web-tool calls recovered (H001–H107). |
| Targeted update | 1 September 2026; actual calls recovered (H108–H114), including literature checks and journal-policy queries. |
| Revision verification | 19 September 2026: retained-candidate reconciliation, targeted source checks and historical-log recovery; no new comprehensive database search. |
| Publication period | No lower publication-date limit; the evidence cutoff remains 1 September 2026. Earlier foundational sources could inform background. |
| Language | English or Chinese; other languages were not eligible. This restriction may omit relevant deployments. |
| Core retrieval sources | AI-assisted web discovery, including site-restricted PubMed/PMC and publisher searches; source-page retrieval and reference following. Four requests for PubMed search-result URLs returned tool-level access errors. |
| Chinese retrieval | Discoverable SinoMed records and publicly accessible Chinese-language/CNKI pages were checked; this was not a complete search of the licensed SinoMed/CNKI databases. |
| Unavailable resources | No institutional exports from Web of Science, Scopus, Embase or full CNKI were available. |
| Reproducibility boundary | Queries, timestamps and returned-source metadata are retained in S3. S2 records candidate decisions. Prior-library provenance and the remaining coverage boundary are described in S14. |

# S3. Recovered PubMed search URL attempts

The four expressions below were decoded from actual PubMed URLs requested at 10:48:34 China time on 28 August 2026 (event H013). All four requests returned tool-level access errors rather than search-result pages. These errors do not mean that PubMed article pages were generally inaccessible. No hit count can be inferred, including zero. The broader Boolean expressions in earlier strategy documents were not verified as successfully executed database queries and are not presented as such here. Actual web-search expressions and timestamps are in Supplementary Table S3, Queries.

## S3.1. Implementation and operating costs

(artificial intelligence[Title/Abstract]) AND (hospital[Title/Abstract]) AND ("implementation cost"[Title/Abstract] OR "operational cost"[Title/Abstract] OR "maintenance cost"[Title/Abstract] OR "total cost of ownership"[Title/Abstract])

## S3.2. Hidden labor and verification

(artificial intelligence[Title/Abstract]) AND (hospital*[Title/Abstract]) AND ("invisible labor"[Title/Abstract] OR "hidden labor"[Title/Abstract] OR "verification burden"[Title/Abstract])

## S3.3. Decommissioning and retirement

(artificial intelligence[Title/Abstract]) AND (hospital*[Title/Abstract]) AND (decommission*[Title/Abstract] OR retire*[Title/Abstract] OR deimplementation[Title/Abstract])

## S3.4. China and implementation

(artificial intelligence[Title/Abstract]) AND (China[Title/Abstract]) AND (hospital*[Title/Abstract]) AND (cost*[Title/Abstract] OR maintenance[Title/Abstract] OR implementation[Title/Abstract])

# S4. Supplementary retrieval and Chinese concepts

Actual retrieval relied on web searches, including domain-restricted PubMed/PMC and publisher queries, followed by source-page checks. Publisher/DOI checks addressed titles, identifiers, publication details and claim-relevant text. Reference following and focused title/identifier searches supplemented discovery. Previously identified records in a retained literature library also informed candidate discovery; X10 is documented in S14.

(人工智能 OR 机器学习 OR 大模型 OR 智能决策支持) AND (医院 OR 医疗机构 OR 县级医院 OR 医共体 OR 医联体) AND (运营 OR 流程 OR 排队 OR 调度 OR 行政 OR 病案编码 OR 设备维护 OR 后勤) AND (成本 OR 经济性 OR 可负担性 OR 资源 OR 工作量 OR 实施 OR 集成 OR 运维 OR 监测 OR 退出)

Additional targeted combinations included 人工智能+县域医院+成本, AI+医共体+运营效率, 智能医院管理+二级医院+运维, and 人工智能+医院+隐性劳动.

The Chinese concept block above is retained strategy documentation, not a verbatim executed query. Supplementary Table S3 contains the actual Chinese-language queries, including domain-restricted CNKI/Wanfang searches and SinoMed record checks. Publicly discoverable results do not constitute complete licensed-database exports.

# S5. Eligibility, screening and extraction

Core eligibility required real hospital/health-system operational use, a reported postdeployment activity or responsibility, and an identifiable bearer in accessible source content. Eligible activities concern human review, integration, upkeep, oversight or transition. Deployment, task-routing and efficiency outcomes alone do not meet this criterion. The original L1 definition included observed work or workflow responsibility, but earlier selection decisions inconsistently excluded small/vendor-linked or uncosted deployments. Reassessment applied this definition explicitly: quantified costs, large samples, favorable outcomes and full-text access were not prerequisites; vendor ties were caveats. This was a revision of selection decisions, not a preregistered procedure. N05, N10, X10, E19 and E20/E21 meet the clarified criterion; E16 remains contextual.

Laboratory-only or hypothetical deployments, pre-procurement/development-only costing, and purely clinical prediction or imaging without a separable operational focus were outside the primary set. Patient-cost outcomes are not AI ownership costs, but do not exclude a report that also documents operational review work. For mixed platforms, the relevant operational activity and bearer must be explicitly described. E16 describes institutional reporting but does not sufficiently establish a separable AI-specific postdeployment task and bearer; it is contextual rather than strict-core evidence. AI-assisted clinical quality control is not treated as surveillance of AI performance.

XW and HW independently screened records for relevance and evidence role. XW extracted setting, task, design, burden domains, bearers, outcome level, deployment identity and limitations; HW checked the extraction. Uncertain classifications or disagreements were referred to a third expert for independent judgment. No agreement coefficient or numerical count of adjudications is reported because these were not available in the retained documentation.

Assessment used available full text or official abstracts/metadata. Metadata verification establishes bibliographic identity, not support for every substantive claim. Extraction and interpretation were limited to accessible content. For Lawrence et al., revision verification was restricted to the official abstract and bibliographic record; unreported details were not assumed absent from the inaccessible full text.

Report families were identified from institution, tool/program and deployment history. UW Health (N01/E24) and UCI (N02/N02b) are paired. The Shanghai randomized report E21 explicitly cites E20 as its preceding cohort and describes the same SCMC/YI TU outpatient program; names XIAO YI and Smart-doctor refer to this linked program history. Different observation periods and samples are preserved. Pairing prevents double counting program-level evidence; it does not assert statistical independence or identical participants.

# S6. Evidence roles and analytic codebook

| **Role** | **Use in this review** |
| --- | --- |
| Primary (“strict-core”) cluster | Twelve burden-focused deployment clusters in main Table 1; mixed-program and abstract-access caveats remain explicit. |
| Companion | Additional report from a primary deployment; informs benefits or mechanisms without adding an independent primary cluster. |
| Adjacent | Clinical prediction or diagnostic imaging monitoring/offboarding; supports analogy, not direct generalization to operational AI. |
| Contextual | China-related implementation/use or screening records that do not establish separable primary burden outcomes. |
| Governance support | Organizational governance/retirement account, not an additional primary operational deployment. |

| **Dimension** | **Category** | **Operational definition** |
| --- | --- | --- |
| Burden domain | Hidden labor | Verification, correction, training, coordination, data upkeep, and workflow adaptation. |
| Burden domain | Monitoring/maintenance | Dashboards, data-quality checks, revalidation, remediation, licensing, hosting, and vendor interfaces. |
| Burden domain | Exit/replacement | Reassessment, termination, data export, migration, fallback, and temporary duplication. |
| Outcome level | L1 | Observed work or workflow responsibility. |
| Outcome level | L2 | Labor value or opportunity cost. |
| Outcome level | L3 | Budget or cash expenditure. |

“Yes” in the CSV indicates a reported activity or requirement, not its incremental duration, net burden, prevalence or causal effect. “No”/“not reported” means that the reviewed material did not supply the relevant evidence, not that the activity never occurred. L3 categories may identify expenditure without a complete monetary total. Vendor relationships are caveats for interpretation, not automatic grounds for exclusion. No formal risk-of-bias instrument was applied; unvalidated internal letter/number grades from the prior working matrix have been removed. L1 includes retained or redistributed responsibilities and does not imply that the work is new, hidden from the source authors, or a net increase attributable to AI.

# S7. Record accounting and cluster map

The original 18-record account comprised nine core/companion reports representing seven deployment clusters (E23; E26; N01/E24; N02/N02b; N03; N04; N08), one secondary-core report (N05), one supplementary implementation report (Lawrence/N10), three adjacent reports (E12/N06/N07), three contextual records (E18/E19/E22) and one governance report (N09). The submitted matrix contained 17 rows and omitted Lawrence, which was already cited and discussed. Restoring its row reconciles 18 records without introducing a new nineteenth record.

Separately, revised selection includes five candidates present in the original documentation: X10/X08 in the exclusion/downgrade ledger and E20/E21/E16 in the earlier evidence matrix. X10 and paired E20/E21 supply core activity-and-bearer evidence. X08 and E16 are contextual and add no core cluster. The inventory has 23 records: 15 primary/companion reports in 12 clusters, three adjacent, four contextual and one governance record. The original seven clusters remain. N05/N10/E19 add three, and X10 plus E20/E21 add two: 7 + 3 + 2 = 12. S15 documents the original decisions, source locations and revised boundaries. These are substantive selection corrections, not simply a counting correction or five newly retrieved publications.

| **Inventory category** | **n** | **Record IDs** |
| --- | --- | --- |
| Primary representatives | 12 | E19, E23, E26, N01, N02, N03, N04, N05, N08, N10, X10, E20 |
| Companions | 3 | E24, N02b, E21 |
| Adjacent | 3 | E12, N06, N07 |
| Contextual | 4 | E16, E18, E22, X08 |
| Governance support | 1 | N09 |
| Total analytic inventory | 23 | 15 primary/companion reports in 12 clusters; eight other analytic records. |

| **Cluster** | **Record IDs** | **Main refs** | **Deployment** |
| --- | --- | --- | --- |
| C1 | E23 | 1 | Taiwan nursing handover |
| C2 | E26 | 12 | Bradford command centre; AI attribution incomplete |
| C3 | N01 + E24 | 6 + 2 | UW Health ambient documentation: monitoring plus trial |
| C4 | N02 + N02b | 10 + 11 | UCI ambient documentation: edits plus interviews |
| C5 | N03 | 18 | German documentation copilot |
| C6 | N04 | 7 | England ambient scribe; hospital and primary-care settings |
| C7 | N08 | 8 | Southeast Asian hospital chatbot lifecycle |
| C8 | N05 | 19 | Israeli pediatric scheduling |
| C9 | N10 | 20 | New York ambient documentation; abstract-accessible |
| C10 | X10 | 13 | Japanese discharge-summary workflow |
| C11 | E19 | 17 | Wenzhou equipment-priority workflow |
| C12 | E20 + E21 | 15 + 16 | Shanghai linked outpatient program; successive periods |

# S8. Record-level selection summary and limits

| **ID / main ref** | **Evidence role** | **Selection rationale / inference boundary** |
| --- | --- | --- |
| E12 / 28 | Adjacent | Real offboarding but radiology is outside strict operational core; 21.4% response and role skew limit inference |
| E18 / Not cited in main text | Contextual screened record | Accessible record describes county consortium implementation but does not establish a specific postdeployment burden activity and its bearer; contextual only. This is not a claim that the full article lacks such evidence. |
| E19 / 17 | Primary cluster representative C11 | Engineers record adoption of recommendations, completion and resource use and retain decision responsibility. AI implementation/personnel costs are missing, but L1 responsibility meets eligibility. Device-maintenance costs are not AI-support costs. |
| E22 / 31 | Contextual | Study concerns individual everyday use rather than formal organizational deployment |
| E23 / 1 | Primary cluster representative C1 | Nurses review correct and confirm outputs; time saved labor value and monthly operating costs are distinguished from cash savings |
| E24 / 2 | Companion C3 | Strong effect evaluation but same deployment as N01; count once and use N01 for monitoring burden |
| E26 / 12 | Primary cluster representative C2 | Staff adaptation and data quality work are observed but attribution to the AI component is incomplete |
| N01 / 6 | Primary cluster representative C3 | Real-time monitoring detected template-associated accuracy fall and triggered redesign training and onboarding work |
| N02 / 10 | Primary cluster representative C4 | 1804 word-level edits across 200 encounters show recurring factual and specificity corrections; time burden not measured |
| N02b / 11 | Companion C4 | Explains why clinicians edit but shares the N02 deployment cluster |
| N03 / 18 | Primary cluster representative C5 | Senior review and revision count are measured; AI did not show a significant between-group increase in review burden |
| N04 / 7 | Primary cluster representative C6 | Integrated deployment required standardization templates and validation; stand-alone use required copying; hospital evidence is mixed with primary care |
| N05 / 19 | Primary cluster representative C8 | Real operational deployment with chief-resident review and adjustments; time/cost quantification is not required; vendor ties are caveats. |
| N06 / 25 | Adjacent | Strong production monitoring ownership evidence but models are clinical predictive tools rather than strict operational AI |
| N07 / 26 | Adjacent | Monitoring of 17 imaging algorithms is adjacent to operational AI; conflict-of-interest information was not fully established in retained extraction. |
| N08 / 8 | Primary cluster representative C7 | Additional data upkeep approvals training license management hosting and manpower were observed; cost-benefit review led to sunset and transition |
| N09 / 29 | Governance support | Reports 10 deployments and 2 retirements but does not quantify postdeployment resource burden |
| N10 / 20 | Primary cluster representative C9 | Official abstract describes an implemented program, multidisciplinary team and continuing technical support. Abstract access limits inference but does not by itself exclude an eligible deployment. |
| X10 / 13 | Primary cluster representative C10 | Responsible therapists verify and correct generated summaries before finalization. Small sample and narrow setting limit inference; they do not negate activity-and-bearer eligibility. |
| X08 / 14 | Contextual | Accessible abstract describes routing patient requests to nursing and nonclinical departments, but does not establish a specific verification, integration, upkeep, oversight or transition activity. Retained as contextual workflow evidence, not a core burden deployment. This is an access-bounded decision, not proof that the full article contains no such activity. |
| E20 / 15 | Primary cluster representative C12 | Backstage physicians review every generated order and manually adjust/approve when needed. Patient waiting-time/fee outcomes do not replace measurement of this responsibility; they are not a reason to exclude L1 evidence. |
| E21 / 16 | Companion C12 | Later trial explicitly links to the earlier SCMC cohort/program and mentions physician review. Counted as companion to E20, not an independent institution or duplicate patient sample. Research-only expert validation is not counted as routine operational burden. |
| E16 / 21 | Contextual | Retained as contextual evidence: routine institutional compilation and review of implementation/utilization summaries are described, but a separable AI-specific postdeployment task and its bearer are insufficiently established for the strict core. This is not evidence of dedicated model surveillance, incremental workload or permanent operational-AI withdrawal. |

S8 summarizes the 23 analytic records. Supplementary Table S2 extends the decision trail to the retained candidate pool, selected background sources and unresolved narrative leads. Its rows are assembled from saved documents. Multiple appearances are linked by known identifiers; unresolved descriptions are not assumed to be unique publications. The coverage boundary is specified in S14.

# S9. Background references, claim mapping and limitations

The manuscript has 31 references: 22 of the 23 analytic inventory records are cited, while E18 remains a supplement-only contextual record; nine background references supply framing. The 15 primary/companion reports represent 12 clusters, not 15 independent deployments. Supplementary Table S2 includes further retained background and out-of-scope candidates not cited in the manuscript.

| **Main reference** | **Background role** |
| --- | --- |
| 3 | Ambient-scribe rapid review; evidence scarcity |
| 4 | AI economic-evaluation systematic review; reporting of implementation/operating costs |
| 5 | Hospital decision-support cost review; adjacent cost categories |
| 9 | LMIC scoping review; transfer limitations |
| 22 | Lifecycle frameworks; background synthesis |
| 23 | MLOps scoping review; operating domains |
| 24 | Expert interviews; monitoring responsibility |
| 27 | NASSS framework; sustainability and abandonment |
| 30 | China diagnostic-imaging implementation context |

| **Main inference** | **Principal evidence** | **Boundary** |
| --- | --- | --- |
| Correction/adaptation persists in some deployments | C1, C2, C4, C6, C7, C10, C11, C12 | Existence/content, not universal net workload increase. |
| Benefits can coexist with continuing review | C1, C3, C5, C8 | No pooled effect; scheduling allocation is distinct from administrative preparation. |
| Monitoring requires ownership and can detect degradation | C3, C2, C7; adjacent N06/N07 | Different monitoring scopes; no transferable total monitoring cost. E16 is contextual and not used to establish AI-model surveillance. |
| Actual operational-AI sunset is documented | C7; adjacent E12 and governance N09 | One direct lifecycle case; no universal exit-cost estimate. |
| Chinese management implications | Core C11–C12; contextual E16/E22; diagnostic-imaging background | Chinese workflow responsibility, not transferable resource-constrained AI lifecycle costs. |

Selection is purposive and may overrepresent burden-visible reports. Recent publications and well-resourced systems predominate. Language/access restrictions, truncated responses and incomplete historical selection records may omit relevant studies. Some evidence is abstract-limited or has mixed-program attribution problems. Chinese operational responsibilities are documented, but resource-constrained hospital lifecycle costs are not established. Cluster frequencies cannot estimate prevalence. The decision framework is proposed, not validated.

# S10. Files, provenance and revision corrections

Supplementary Table S1 contains 23 analytic inventory rows with stable IDs, current reference numbers, evidence roles, clusters, burden fields and access limits. S2 contains the retained-candidate decision trail. Inherited checks are not described as a new complete full-text audit. Targeted checks on 19 September assessed N05/N10/E18 and the newly reconsidered X08/X10/E19/E20/E21/E16; E17 was checked to assess the boundary of mixed-program evidence. S2 provides full citations and reference-number links for every source cited in the main text. Other rows retain an identifier or a clearly labeled unresolved description; bibliographic enrichment is not presented as new full-text verification. Source-location checks for the selection explanations were supplemented on 20 September; these did not extend the publication cutoff or constitute a new comprehensive search.

Corrections include the N10 matrix omission, reassessment of earlier selection decisions and retention of E16 and X08 as contextual evidence; consistent inclusion of qualitative, small-sample and abstract-accessible activity-and-bearer evidence; additional paired Shanghai reports; removal of the claim that no mainland-China core deployment was identified; and alignment of reference numbers. Earlier corrections to publication year, qualitative design labels, scheduling interpretation and unvalidated quality grades are retained. No evidence published after the 1 September cutoff was added. Original strategy and staged files remain unchanged.

Verification links: Gilad et al., https://www.jmir.org/2026/1/e88340/ ; Lawrence et al., https://link.springer.com/article/10.1007/s11606-026-10448-w . The full bibliographic entries are in the main reference list.

# S11. Search expression source and date crosswalk

This crosswalk now uses recovered execution records. H identifiers refer to the chronological calls in Supplementary Table S3; timestamps are retained there. Earlier strategy documents remain preserved separately but are not substituted for actual query execution. The representative expressions below are copied verbatim from recorded web queries; the complete recovered query list is supplied in S3.

| Date / record | Source and expression | Documentation boundary |
| --- | --- | --- |
| 28 Aug 2026 direct PubMed attempts | H013; the four URL-decoded expressions in S3.1–S3.4 | Access errors for all four URLs; no successful database result set or hit count. |
| 28 Aug 2026 discovery and verification | H001–H107; see Queries, Events and ReturnedPages in Supplementary Table S3 | Includes topical, title/identifier and journal-policy searches; some response text is truncated. |
| 28 Aug 2026 Chinese discovery | Examples H012, H029, H031, H032 and H065 | Public-web CNKI/Wanfang/SinoMed discovery or checking, not a licensed-database export. |
| 1 Sep 2026 update | H108–H114; H111 contains four actual topical update queries | H112–H114 check the Lawrence article; batched query results cannot be attributed to one query. |
| 19 Sep 2026 revision work | Source verification, retained-candidate reconciliation and recovery of the historical session | No rerun is represented as an original search, and the evidence cutoff remains 1 September. |

H023-Q1 (2026-08-28 11:03:58, China time): site:pubmed.ncbi.nlm.nih.gov hospital artificial intelligence implementation cost economic evaluation operational workflow

H053-Q1 (2026-08-28 18:01:54, China time): site:pubmed.ncbi.nlm.nih.gov hospital artificial intelligence post-deployment monitoring maintenance workflow staff workload real-world

H111-Q1 (2026-09-01 16:33:50, China time): site:pubmed.ncbi.nlm.nih.gov hospital artificial intelligence implementation monitoring workload maintenance 2026

H029-Q1 (2026-08-28 11:05:04, China time): site:cnki.net 医院 人工智能 运营管理 成本 实施 县域

English/Chinese eligibility and no lower publication-date limit are supported by the retained strategy, not assumed to be filters applied identically by each search engine. S3 documents execution and returned pages; S2 documents retained-candidate decisions.

# S12. Identifiable source for contextual record E18

张楚柔, 饶远立, 马海燕, 莫尔淳, 许仕杰. 粤北县域“AI+中医院医共体”高质量建设的创新与实践. 新中医. 2026;58(2). SinoMed record 2026200239. https://www.sinomed.ac.cn/article.do?ui=2026200239 (indexed record checked 19 September 2026).

The source was verified through the official indexed SinoMed metadata and accessible abstract excerpt, not the full article. The year is also supported by the retained bibliography and 2026 issue metadata. Page range and DOI were not verified. This contextual record is not evidence of measured postdeployment burdens and is not added to the main manuscript references. Its limited accessible content does not establish that the full article contains no eligible burden evidence.

# S13. Retained-candidate reconciliation and provenance

Supplementary Table S2 contains 63 retained candidate/lead entries after merging known repeated appearances, including unresolved or grouped leads. This number is a present-day documentation count, not the original number of retrieved, screened or unique published studies. The 23-row analytic inventory is a selected subset; background and non-core decisions remain visible in S2.

The source chain includes the original strategy documents, staged evidence matrices and representative exclusion ledger, plus the recovered 28 August and 1 September web-tool calls and saved result metadata. It also includes the earlier literature library read on 28 August, which contains the X10 record. Original files remain unchanged. Supplementary Table S3 links timestamps and returned source pages to stable candidate identifiers without manufacturing missing selection decisions.

The initial exploratory stage did not use independent dual screening. The independent screening and extraction-checking procedure described in S5 applies to the later review work.

Known alias links: SC-C01/E08, SC-C03/E01, SC-C05/E03, SC-C06/E02, SC-C07/E04, SC-C08/E09, SC-C11/E15, SC-C12/E16 and SC-C16/E19. SC prefixes distinguish competitor-matrix IDs from deployment clusters. X01 is a separate preprint-version entry linked to E11, not another deployment. L01–L03 and X06/X07 remain unresolved descriptions in the decision ledger. Retrieval provenance in S3 does not itself resolve all candidate identities.

Important scope limits: Retained as contextual evidence: routine institutional compilation and review of implementation/utilization summaries are described, but a separable AI-specific postdeployment task and its bearer are insufficiently established for the strict core. This is not evidence of dedicated model surveillance, incremental workload or permanent operational-AI withdrawal. E20/E21 patient expenditure is not hospital AI cost. E19 equipment-maintenance expenditure is not AI maintenance expenditure. X08 supplies contextual evidence on staff-task routing from an abstract; it is not a core burden deployment or proof of net labor saving. X10 supports therapist verification/correction despite its small sample.

# S14. Recovered execution archive

Supplementary Table S3 contains five sheets: Readme, Queries, Events, ReturnedPages and CandidateLinks. We recovered 114 web-tool calls from the saved research session: 107 on 28 August and seven on 1 September. Of these, 77 calls contain 272 actual web queries. Four failed PubMed search-URL attempts are separately labeled. These counts include journal-policy and source-verification queries and are not study-selection totals.

The archive contains 1,349 returned page occurrences, representing 958 distinct URLs after terminal-slash normalization. Repeated retrievals, website pages, preprints and journal-policy pages are retained as returned; the number is not a count of unique publications. Eighteen response texts carry truncation markers. Saved result metadata supplied five additional page occurrences absent from the text headers. Omitted article text was not reconstructed.

Each query is linked to its original batch, time and returned source metadata. Batched results cannot be attributed to individual queries. CandidateLinks matches 54 of 63 retained candidate/lead entries, including 22 of 23 analytic records, to returned web-source metadata. The remaining analytic record, X10 (Nakano; PMID 42326256; DOI 10.7759/cureus.109247), is present in a retained PubMed export whose search log is dated 25 August 2026. Its saved query membership comprises broad workflow, implementation/evaluation, documentation/scribe and negative-outcome/cost modules. The 28 August session records reading that library and registering X10 in the original exclusion/downgrade ledger. This establishes a prior-library source route, not an additional web-return match or original core-inclusion decision. The earlier library concerned a separate review and its search totals are not used as this review’s screening denominator.

The documentation establishes historical search activity and source provenance. The retained records do not provide a complete list of retrieved publications, a complete historical exclusion list or the original screening total. The review remains a focused, purposively selected narrative synthesis and does not claim exhaustive coverage. Recorded queries and dates are historical, not a new search conducted during revision.

The public archive includes query text, dates, result titles/URLs, event identifiers, source-line locators and request/response hashes. Full conversations, local personal paths and article full text are excluded. A task-specific extract of original search calls, responses and saved result metadata is retained separately for author verification. The archive does not imply that the authors had already reviewed every newly recovered record before this revision.

## S15. Original accounting and evidence for selection changes

The original roles below explain the submitted 18-record/seven-cluster account. They are historical classifications, not a claim that all earlier decisions remain appropriate. Stable record IDs identify sources across Tables S1–S3. Current cluster IDs C11 and C12 denote Wenzhou and Shanghai, respectively; X08 has no core cluster.

| **Original role** | **Records** | **IDs** |
| --- | --- | --- |
| Core representatives | 7 | E23, E26, N01, N02, N03, N04, N08 |
| Companions | 2 | E24, N02b |
| Secondary core | 1 | N05 |
| Supplementary implementation | 1 | N10 (Lawrence) |
| Adjacent | 3 | E12, N06, N07 |
| Contextual | 3 | E18, E19, E22 |
| Governance | 1 | N09 |
| Total | 18 | Nine core/companion reports represent seven clusters |

The following source locations explain why the revised roles differ. Responsibility evidence does not establish incremental workload, net cost, permanent support requirements or lifecycle affordability. Source access limits remain record-specific in S1–S2.

| **Record and role change** | **Reported activity and source location** | **Interpretive boundary** |
| --- | --- | --- |
| N05: secondary core → primary C8 | Gilad et al., Methods, postimplementation scheduler description: chief residents configure constraints, review drafts and adjust assignments. | Explicit human review. Vendor relationships limit interpretation; edit logs and preparation time were not available. |
| N10: supplementary implementation → primary C9 | Lawrence et al., official abstract, Program Evaluation and Discussion: continuing technical support, training resources and integration needs. | Abstract-supported responsibilities only. Matrix omission and change of analytic role are separate corrections. |
| E19: contextual → primary C11 | Ye et al., section 2.6: engineers record recommendation adoption, completion, response and resource use during deployment. | Observed engineering responsibility, not AI-support expenditure. Evaluation-period recording cannot establish a permanent additional task. |
| X10: excluded core → primary C10 | Nakano and Imura, Materials and Methods, AI-assisted workflow description: responsible therapists verify, correct and finalize summaries. | Supports L1 verification work despite small sample. It does not demonstrate net workload increase. |
| E20/E21: excluded core → C12 representative/companion | Li et al. 2021, AI-assisted process description: backstage doctors review and approve orders. Li et al. 2022, Introduction/Methods and Discussion: prior-program link and doctor review. | Same institutional program, successive study periods. Patient fees are not hospital AI costs; research-only expert validation is not counted as routine work. |
| X08: downgraded candidate → contextual | Gentil et al., accessible official abstract, Methods/Results: patient requests routed among nursing and nonclinical teams. | Routing alone does not establish a specific burden-domain activity. Full text was not accessible; do not infer absence from the full article. |
| E16: excluded core → contextual | Tian et al., retained implementation/utilization reporting extract. | No sufficiently separable AI-specific postdeployment activity and bearer established. No core cluster added. |
